# Actinic Cheilitis: A Systematic Review and Meta-Analysis of Interventions, Treatment Outcomes, and Adverse Events

**DOI:** 10.3390/biomedicines13081896

**Published:** 2025-08-04

**Authors:** Matthäus Al-Fartwsi, Anne Petzold, Theresa Steeb, Lina Amin Djawher, Anja Wessely, Anett Leppert, Carola Berking, Markus V. Heppt

**Affiliations:** 1 Department of Dentistry, Uniklinikum Erlangen, Friedrich-Alexander-Universität Erlangen-Nürnberg (FAU), 91054 Erlangen, Germany; 2 Department of Dermatology, Uniklinikum Erlangen, Friedrich-Alexander University Erlangen-Nürnberg, 91054 Erlangen, Germany; anne.petzold@uk-erlangen.de (A.P.); theresa.steeb@uk-erlangen.de (T.S.); lina.djawher@uk-erlangen.de (L.A.D.); anja.wessely@uk-erlangen.de (A.W.); anett.leppert@uk-erlangen.de (A.L.); carola.berking@uk-erlangen.de (C.B.); 3 Comprehensive Cancer Center (CCC) Erlangen-EMN, CCC Alliance WERA, 91054 Erlangen, Germany; 4 Bavarian Cancer Research Center (BZKF), 91054 Erlangen, Germany; 5 Dermpath München, Laboratory for Dermatopathology, Oral Pathology and Molecular Pathology, 80335 Munich, Germany

**Keywords:** cheilitis, actinic, precancerous conditions, aminolevulinic acid, laser therapy, diclofenac/therapeutic use, imiquimod/therapeutic use, meta-analysis as topic, treatment outcome

## Abstract

**Introduction:** Actinic cheilitis (AC) is a common precancerous condition affecting the lips, primarily caused by prolonged ultraviolet radiation exposure. Various treatment options are available. However, the optimal treatment approach remains a subject of debate. **Objective:** To summarize and compare practice-relevant interventions for AC. **Materials and Methods:** A pre-defined protocol was registered in PROSPERO (CRD42021225182). Systematic searches in Medline, Embase, and Central, along with manual trial register searches, identified studies reporting participant clearance rates (PCR) or recurrence rates (PRR). Quality assessment for randomized controlled trials (RCTs) was conducted using the Cochrane Risk of Bias tool 2. Uncontrolled studies were evaluated using the tool developed by the National Heart, Lung, and Blood Institute. The generalized linear mixed model was used to pool proportions for uncontrolled studies. A pairwise meta-analysis for RCTs was applied, using the odds ratio (OR) as the effect estimate and the GRADE approach to evaluate the quality of the evidence. Adverse events were analyzed qualitatively. **Results:** A comprehensive inclusion of 36 studies facilitated an evaluation of 614 participants for PCR, and 430 patients for PRR. Diclofenac showed the lowest PCR (0.53, 95% confidence interval (CI) [0.41; 0.66]), while CO_2_ laser showed the highest PCR (0.97, 95% CI [0.90; 0.99]). For PRR, Er:YAG laser showed the highest rates (0.14, 95% CI [0.08; 0.21]), and imiquimod the lowest (0.00, 95% CI [0.00; 0.06]). In a pairwise meta-analysis, the OR indicated a lower recurrence rate for Er:YAG ablative fractional laser (AFL)-primed methyl-aminolevulinate photodynamic therapy (MAL-PDT) (Er:YAG AFL-PDT) compared to methyl-aminolevulinate photodynamic therapy (MAL-PDT) alone (OR = 0.22, 95% CI [0.06; 0.82]). The CO_2_ laser showed fewer local side effects than the Er:YAG laser, while PDTs caused more skin reactions. Due to qualitative data, comparability was limited, highlighting the need for individualized treatment. **Conclusions:** This study provides a complete and up-to-date evidence synthesis of practice-relevant interventions for AC, identifying the CO_2_ laser as the most effective treatment and regarding PCR and imiquimod as most effective concerning PRR.

## 1. Introduction

Actinic cheilitis (AC) was first described by DuBreuilh in 1886 during the Third International Congress of Dermatology [1]. AC is considered the equivalent of actinic keratosis (AK) on the vermilion border of the lips [2,3]. The condition predominantly affects the lower lip and is most commonly observed in fair-skinned males aged 40 to 80 years [1,4], particularly following prolonged ultraviolet (UV) radiation exposure [2,3]. Chronic UV exposure leads to the accumulation of DNA damage, particularly the formation of cyclobutane pyrimidine dimers and 6-4 photoproducts. Mutations in tumor suppressor genes such as *TP53*, as well as disturbances in the Notch signaling pathway, promote dysregulated keratinocyte proliferation and impair apoptosis—mechanisms central to carcinogenesis in AC. AC is regarded as a precursor to squamous cell carcinoma of the vermilion (SCC), with studies reporting a progression rate of 3.2–16.9% to invasive SCC [3,5]. Lip SCCs exhibit a higher metastatic potential compared to cutaneous SCCs. Consequently, timely diagnosis and appropriate management of AC are critical [3,6]. Beyond its oncologic potential, AC may cause significant functional and aesthetic impairments such as lip rigidity, speech disturbances, and reduced quality of life—especially in advanced stages.

Preventive strategies—including consistent UV protection, smoking cessation, and regular monitoring—are particularly crucial in high-risk populations such as immunosuppressed individuals or those with chronic occupational sun exposure.

Numerous therapeutic approaches have been developed for the treatment of AC [7]. However, treatment of lesions on the lower lip comes with unique challenges. Common therapies used for AK often demonstrate reduced efficacy and are more challenging, and oftentimes off-label, to administer in this anatomic region. Furthermore, these treatments may be associated with a higher rate of adverse effects [2,3].

Treatment options for AC range from non-invasive to invasive modalities. Despite these advancements, there remains ongoing debate regarding the most suitable treatment approach. Compared to previous systematic reviews [8,9], which provided early comparative insights into the efficacy of surgical and conservative treatments, this study offers a more comprehensive and up-to-date assessment of treatment approaches for AC. It incorporates additional data sources, includes non-randomized studies for greater completeness, and applies the GRADE approach to evaluate the quality of evidence—an aspect not addressed in earlier reviews. Furthermore, this review highlights the increasing relevance of combination therapies, such as ablative fractional Er:YAG laser followed by photodynamic therapy (AFL-PDT) [10,11], which were not adequately addressed in prior analyses despite promising results in reducing recurrence rates. As such, this work provides a more nuanced and methodologically robust foundation for understanding current therapeutic strategies in AC.

## 2. Materials and Methods

### 2.1. Protocol, Registration, Eligibility Criteria, and Outcomes of Interest

**Figure 1:**
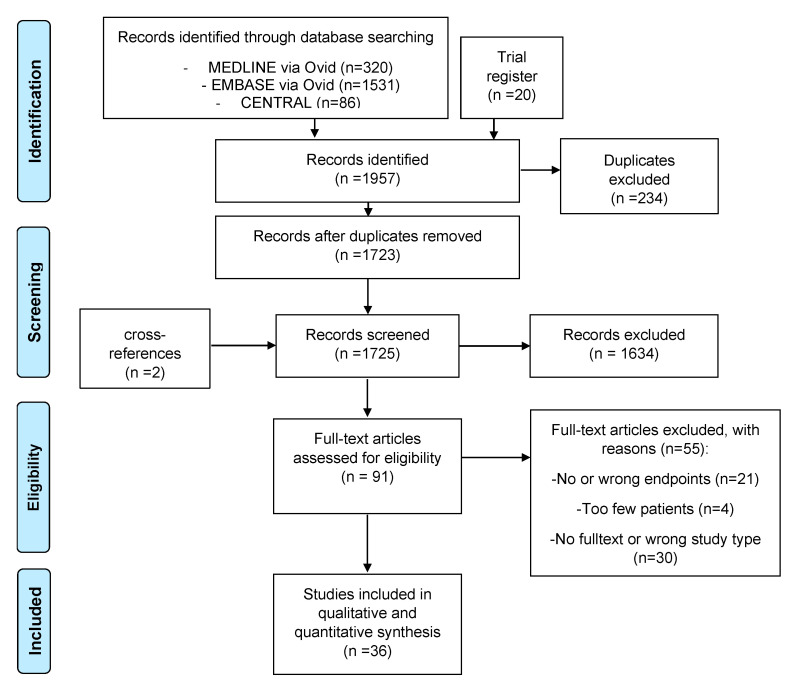
PRISMA flowchart showing the selection process for study inclusion according to the Preferred Reporting Items for Systematic Reviews and Meta-Analysis (PRISMA).

This study was conducted following the Preferred Reporting Items for Systematic Reviews and Meta-Analyses (PRISMA) [12] (Figure 1 and Figure S1). The study protocol was pre-specified and registered in the PROSPERO International Prospective Register of Systematic Reviews (CRD42021225182; registration date: 1 October 2020). The eligibility criteria and predefined outcomes of interest are summarized in Table 1. The aim was to analyze data from patients with AC who received any relevant treatment, including both surgical and non-surgical approaches (5-fluorouracil (5-FU), ingenol mebutate (IMB), imiquimod, diclofenac, ablative laser therapies (CO_2_ laser, Er:YAG laser), and photodynamic therapy (PDT) such as 5-aminolevulinic acid (ALA) and methyl-aminolevulinate (MAL), PDT with red light, MAL-PDT with daylight, and Er:YAG ablative fractional laser (AFL)-combined MAL-PDT (Er:YAG AFL-PDT). Records were included from randomized controlled trials (RCTs), controlled clinical trials, non-controlled clinical trials, retrospective studies, and larger case series (*n* > 5) that reported the outcomes of interest. There were no restrictions regarding language. These efficacy outcomes were as follows: (i) participant clearance rates (PCR), defined as complete (100%) clearance of the entire lip; and (ii) participant recurrence rates (PRR), defined as the percentage of participants with recurrent lesions after complete clearance. Furthermore, adverse events were extracted and summarized qualitatively. Exclusion criteria were as follows: Studies with a sample size ≤ 5 patients (e.g., small case reports or very small case series); studies not reporting at least one of the pre-specified efficacy or safety outcomes; studies focusing on actinic keratosis without separate analysis for actinic cheilitis; and in vitro or animal studies.

### 2.2. Literature Search and Study Selection

A comprehensive search of the electronic databases MEDLINE, Embase, and the Cochrane Library (CENTRAL) was conducted to identify all potentially relevant studies published up to 27 August 2024. The full search strategy is provided in Supplementary Table S1.

Two reviewers (MA, AP) independently screened titles and abstracts for eligibility. For studies deemed potentially relevant, full-text articles were retrieved and assessed against the predefined inclusion and exclusion criteria. Whenever discrepancies arose, resolution was achieved by discussion with a third independent author (MVH). Although the literature search covered publications up to 27 August 2024, no eligible interventional studies published after 2021 met the inclusion criteria. Several more recent articles were excluded due to reasons such as being review articles, not reporting outcomes of interest, or including fewer than six patients.

### 2.3. Data Collection, Synthesis, and Management

For each included study, detailed information on the study design (e.g., retrospective, prospective, RCT), population characteristics (number of reported patients, age, sex, continent), intervention type and specifics (e.g., agent, dose, duration, delivery method), comparator (if applicable), and reported outcomes, including PCR, PRR, and adverse events were collected. Data were independently extracted by two authors (MA, AP) using a standardized and piloted data extraction form. Since the literature search did not yield a sufficient number of comparative studies, conducting a network meta-analysis was not feasible. A standard pairwise meta-analysis could only be performed for two RCTs [10,11] that investigated the same interventions.

For all other prospective and retrospective non-comparative studies, the reported clearance and recurrence rates were extracted, and a random-effects meta-analysis of proportions was conducted for each outcome. This was implemented using a generalized linear mixed model (GLMM), with treatment modalities analyzed separately in defined subgroups. A detailed explanation of the GLMM approach, including model specification, link functions, and distributional assumptions, is provided in Supplementary Material S1 [13,14].

We aimed to apply an intention-to-treat (ITT) principle by including all participants enrolled in each study, regardless of outcome availability. However, given the nature of the included studies—many of which were retrospective and only reported data for participants with complete follow-up—we used a modified intention-to-treat (mITT) approach. Specifically, if outcome data were entirely missing for some participants, these cases were excluded from the outcome-specific analysis. In studies reporting only per-protocol populations, data were included as reported. All statistical analyses were performed in RStudio (Version 2022.07.2+576; https://www.rstudio.com/(accessed on 1 September 2024) using the “meta” (version 8.0-1) and “metafor” (version 4.6-0) packages. Results are presented with corresponding 95% confidence intervals (CIs).

### 2.4. Assessment of Study Quality, Heterogeneity, Sensitivity Analysis, and Risk of Bias

To evaluate the methodological quality of the studies, the Cochrane Risk of Bias tool (RoB 2) for RCTs was employed. For uncontrolled studies, the Quality Assessment Tool for Before-After (Pre-Post) Studies with No Control Group was used, developed by the National Heart, Lung, and Blood Institute (NHLBI) (available at https://www.nhlbi.nih.gov/health-topics/study-quality-assessment-tools (accessed on 10 January 2025). This tool comprises 12 items that assess key methodological domains, including the following:

(1) sample selection and representativeness;
(2) clarity and reliability of exposure and outcome measures;
(3) temporal sequence of intervention and outcome;
(4) presence and handling of confounding variables;
(5) appropriateness of statistical analysis; and
(6) completeness and transparency of result reporting.

The NHLBI tool does not define specific thresholds for quality scores; however, studies were categorized based on their performance: good (≥9 criteria fulfilled), fair (5–8 criteria fulfilled), or poor (≤4 criteria fulfilled). The quality assessment was conducted independently by two authors (MA, AP), with discrepancies resolved through discussion until a consensus was reached. Additionally, the quality of the evidence from the pairwise meta-analysis was evaluated with the GRADE approach, using GRADEpro GDT (version November 2024) software (https://www.gradepro.org/, accessed on 18 November 2024) [10,11].

The variability between studies was assessed using the I^2^ statistic, which quantifies the proportion of variability in effect estimates attributable to differences in true effects rather than random chance [15]. The I^2^ values are interpreted as low (25–50%), moderate (50–75%), or high (>75%) heterogeneity. Sensitivity analysis was not performed in this study due to the current lack of functionality for such analyses in the GLMM meta-analytical approach. To assess small-study bias, including potential publication bias, a funnel plot was generated for each outcome.

## 3. Results

### 3.1. Identification and Characteristics of Included Studies

The initial literature search yielded 1957 records. After removal of duplicates and screening of titles and abstracts, 91 full-text articles were assessed for eligibility. Of these, 55 were excluded based on the predefined criteria, with detailed reasons provided in Figure 1. Ultimately, 36 studies met the inclusion criteria and were included in the final analysis. There were 15 retrospective and 17 prospective single-arm studies, and four RCTs. The methodological quality of the uncontrolled studies was rated as good for n = 6, while n = 20 studies were rated as fair, and n = 6 studies achieved only poor quality in the rating with the NHLBI assessment tool. The four RCTs assessed using the RoB 2 tool were all classified as having a high risk of bias. Histopathological confirmation occurred before treatment was reported in 33 out of 36 studies; however, in 9 of these, not all participants underwent biopsy before therapy. Post-treatment histological assessment was documented in 22 studies, with 6 of them lacking consistent post-treatment biopsy for all patients.

Patient and study characteristics of all included studies listed by treatment modality are summarized in Table S2. Finally, 614 patients were evaluable for PCR, 430 for PRR, and 54 patients were included in the comparative two-armed meta-analysis, based on the studies by Ko (2014) and Choi (2015) [10,11].

### 3.2. Efficacy Estimates Derived from Uncontrolled Studies

#### 3.2.1. Participant Clearance Rate

**Figure 2:**
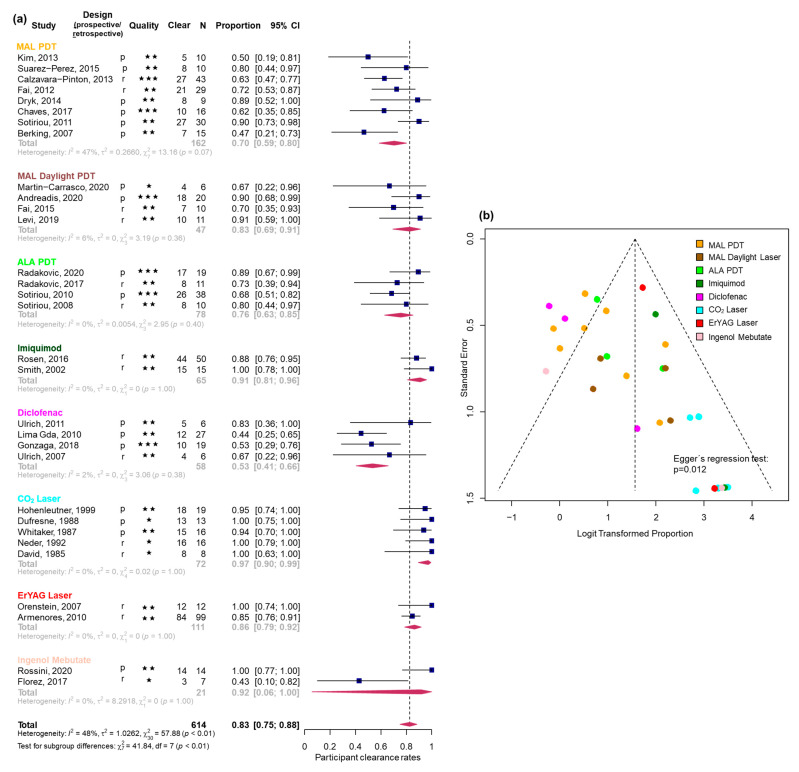
(**a**) Forest plot for the pooled participant clearance rates (PCR). Each study design is indicated by ‘p’ for prospective and ‘r’ for retrospective, while study quality is represented by stars: one star (★) for poor quality, two stars (★★) for fair quality, and three stars (★★★) for good quality (assessed with the quality control tool developed by the National Heart, Lung, and Blood Institute). N represents the number of participants in each study for whom clearance data were available. The diamond represents the summary proportion from each treatment group and at the bottom is the summary proportion of all uncontrolled studies. The width of the line extending from each square and the diamond width represent the 95% confidence interval (CI). (**b**) Funnel plot of all uncontrolled studies included for PCR color-coded by treatment groups. Refs. [1,2,3,7,16,17,18,19,20,21,22,23,24,25,26,27,28,29,30,31,32,33,34,35,36,37,38,39,40,41,42] are the presented studies.

For PCR, six good-quality [1,3,16,17,18,19], 20 fair-quality [2,7,20,21,22,23,24,25,26,27,28,29,30,31,32,33,34,35,36,37], and five poor-quality [38,39,40,41,42] studies were available. The pooled PCR for all studies was 0.83 (95% CI [0.75; 0.88], I^2^ = 48%) (Figure 2a). The highest PCRs were observed for CO_2_ laser and imiquimod, followed by Er:YAG laser, daylight PDT, and red-light PDT. Diclofenac showed the lowest PCR. Due to a wide confidence interval, the results for IMB could not be reliably ranked. Detailed estimates are provided in Table 2. While heterogeneity was low to moderate across all subgroups (e.g., I^2^ = 0–47%), the interpretation of I^2^ values should be approached with caution, particularly in subgroups with only two studies, where the estimate is statistically unstable. The funnel plot revealed significant asymmetry (Egger’s regression test: *p* = 0.012, Figure 2b).

#### 3.2.2. Participant Recurrence Rate

**Figure 3:**
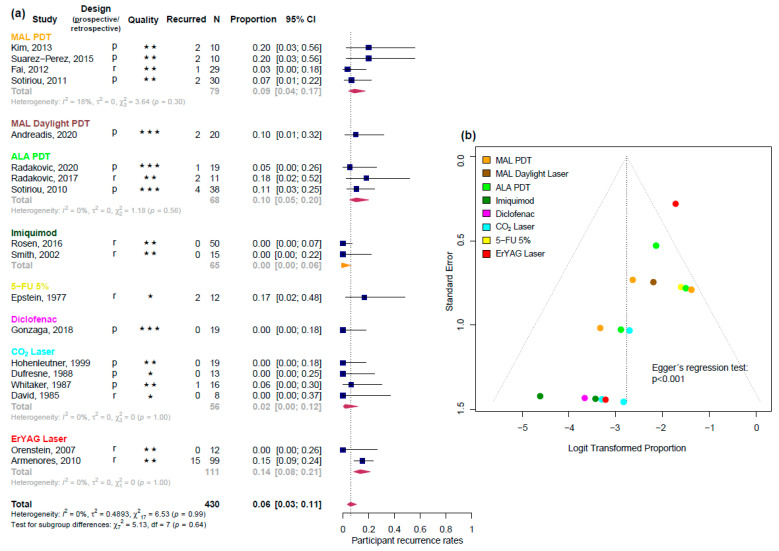
(**a**) Forest plot for the pooled participant recurrence rates (PRR). Each study design is indicated by ‘p’ for prospective and ‘r’ for retrospective, while study quality is represented by stars: one star (★) for poor quality, two stars (★★) for fair quality, and three stars (★★★) for good quality (assessed with the quality control tool developed by the National Heart, Lung, and Blood Institute). N represents the number of participants in each study for whom recurrence data were available. As the meta-analytical method was used for proportions, the generalized linear mixed model (GLMM) was used with one exception in the subgroup of Imiquimod (orange diamond). Here, the binomial distribution with homogeneity assumption was used to calculate the pooled proportion because all study arms showed zero recurrences so that it was not possible to calculate a random effects model within GLMM. The diamond represents the summary proportion from each treatment group and, at the bottom, the summary proportion of all uncontrolled studies. The width of the line extending from each square and the diamond width represent the 95% confidence interval (CI). (**b**) Funnel plot of all studies included for PRR is color-coded by treatment groups. Refs. [3,7,17,18,19,20,21,22,24,27,29,30,34,35,36,39,41,43] are the presented studies.

The analysis of PRR enclosed four good-quality [3,17,18,19], 11 fair-quality [7,20,21,22,24,27,29,30,34,35,36], and three poor-quality [39,41,43] studies. The pooled PRR was 0.06 (95% CI [0.03; 0.11], I^2^ = 0%) (Figure 3a). Imiquimod and CO_2_ laser showed the lowest PRRs, while Er:YAG laser had the highest. Other interventions such as PDT and diclofenac demonstrated intermediate values. Treatments evaluated in single studies could not be reliably ranked (Table 3). While I^2^ values were low or not assessable due to the limited number of studies per subgroup, this should not be interpreted as evidence of homogeneity. I^2^ estimates are inherently unstable in meta-analyses with only a few studies and should, therefore, be interpreted cautiously. Moreover, the reliability of recurrence estimates is further constrained by inconsistent follow-up intervals, a lack of standardized recurrence definitions, and limited reporting in many studies. Most included follow-up periods ranged from 12 to 18 months, with a few extending up to 5 years. Yet, the exact timing of recurrences was seldom specified. Only a small number of studies [17,24,35] reported recurrences at defined intervals like 6 or 12 months. Several studies lacked sufficient follow-up data altogether, which weakens confidence in comparative recurrence estimates. The funnel plot indicated significant asymmetry (Egger’s regression test: *p* < 0.001) (Figure 3b).

### 3.3. Efficacy Estimates Derived from RCTs

**Figure 4:**
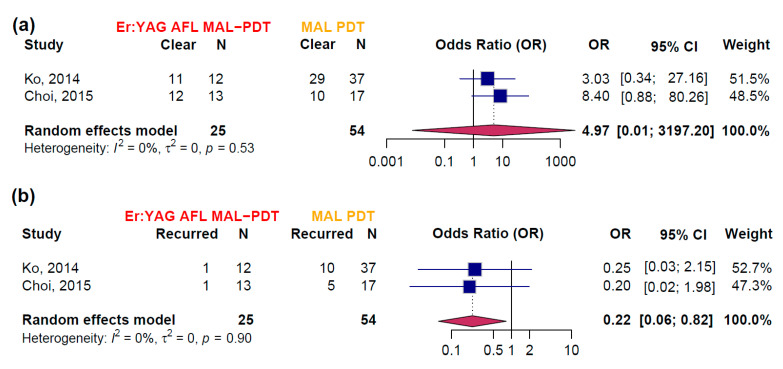
Pairwise meta-analysis comparing treatment outcomes between Er:YAG AFL-PDT and MAL-PDT alone. N represents the number of participants in each study for whom outcome data were available. (**a**) Analysis of PCR, showing odds ratios (OR) with 95% confidence intervals (CI) for studies “Ko, 2014” [10] and “Choi, 2015” [11]. The pooled OR suggests a higher clearance rate in the Er:YAG AFL-PDT group, though with a wide confidence interval. (**b**) Analysis of PRR, showing ORs with 95% CIs for studies “Ko, 2014” [10] and “Choi, 2015” [11]. The pooled OR indicates a lower recurrence rate for Er:YAG AFL-PDT compared to MAL-PDT alone, with a statistically significant effect (OR = 0.22, 95% CI [0.06; 0.82]). Heterogeneity is minimal for both analyses (I^2^ = 0%).

Pairwise meta-analysis was possible for the comparison of Er:YAG AFL-PDT versus MAL-PDT with red light for the outcomes PCR and PRR (Figure 4). The combined OR for PCR was 4.97 (95% CI [0.01; 3197.20]) in favor of Er:YAG AFL-PDT. However, the wide confidence interval reflects considerable uncertainty due to limited data, as only two studies [10,11] were included. Heterogeneity was low (I^2^ = 0%, *p* = 0.53), suggesting consistency between the studies. The OR for PRR was 0.22 (95% CI [0.06; 0.82]) in favor of Er:YAG AFL-PDT. The estimate was based on the same two studies, with low heterogeneity (I^2^ = 0%, *p* = 0.90), indicating consistent results across studies. Both studies exhibited a high risk of bias and the certainty of evidence for the meta-analysis was moderate according to GRADE (Table S3).

Two RCTs included in this systematic review could not be incorporated into a two-arm meta-analysis because they had more than two treatment arms and did not evaluate the same treatment comparisons. In the RCT conducted by Robinson et al. [44], 5-FU 5% was compared with chemical peeling, lip shave, and CO_2_ laser. There were five recurrences (out of 10 patients) in the 5-FU 5% group, seven recurrences (out of 10 patients) in the chemical peel group, and no recurrences in either the lip shave or CO_2_ laser groups. In the study by Husein ElAhmed et al. [45], IMB was compared with imiquimod and diclofenac. The clearance rate with imiquimod (5 out of 10 patients achieved complete clearance) was statistically similar to IMB (4 out of 10 patients achieved complete clearance) (*p* = 0.22) but significantly higher than with diclofenac (2 out of 10 patients achieved complete clearance) (*p* = 0.03).

### 3.4. Adverse Events

Table S4 qualitatively summarizes the side effects of the interventions and their reported frequencies, based on clinical data from the included studies. The table highlights the potential risks of each treatment, with side effects specifically linked to the corresponding interventions. Most adverse events were reported with ALA-PDT with red light, Er:YAG laser, and MAL-PDT with red light.

## 4. Discussion

This study provides an up-to-date and complete overview of reported practice-relevant interventions for AC. This work complements previous reviews by not only confirming the results but also incorporating additional data sources not considered in previous analyses. For the sake of completeness, non-randomized studies were included. In addition, IMB is still included, although it has no longer been approved in the EU since 2020 [46] due to safety concerns.

Compared to previous systematic reviews by Trager et al. [9], Lai et al. [47], Salgueiro et al. [48], and Bakirtzi et al. [8], the present study provides a more comprehensive evaluation of treatment options for AC. A total of 36 studies were included—more than prior reviews—and a quantitative data synthesis of both clearance and recurrence rates was conducted. Furthermore, the study quality of RCTs was assessed with the GRADE approach, which was not performed before and thus brings novelty to this study.

In contrast to earlier reviews that often favored surgical options—postulating complete cures with low recurrence and tolerable side effects [9]—this analysis emphasizes the effectiveness of conservative treatments. In particular, the findings herein confirm previous evidence supporting CO_2_ laser [8,9,49] and imiquimod [50] as highly effective interventions, with strong primary data from RCTs by Robinson [44] and Husein ElAhmed [45]. Further, these findings were extended through the inclusion of newer modalities such as Er:YAG AFL-assisted MAL-PDT and a detailed evaluation of adverse events.

The present study addresses the currently heterogeneous treatment patterns for AC and provides the most up-to-date and clinically relevant synthesis to guide therapeutic decision-making. These results should inform future guidelines and underscore the need for additional high-quality RCTs, particularly given the clinical challenges and patient variability associated with AC.

For the outcome PCR, the highest clearance rates were reported for CO_2_ laser and the lowest ones for diclofenac. IMB could not be reliably classified into a treatment ranking due to a wide confidence interval. For the outcome PRR, imiquimod achieved the lowest rates, followed by CO_2_ laser. MAL-PDT and ALA-PDT with red light showed slightly higher rates, while Er:YAG laser showed the highest PRR. Diclofenac, MAL daylight PDT, and 5-FU 5% were not reliably ranked due to the limited number of studies. The pairwise meta-analysis examined the efficacy of Er:YAG AFL-PDT compared to MAL-PDT for both PCR and PRR, revealing a trend towards higher clearance with Er:YAG AFL-PDT, but with high uncertainty due to limited data and a high risk of bias in the included studies. Heterogeneity was low, indicating consistency between the two studies.

The analysis of side effects highlights that Er:YAG laser, although widely used in dermatologic practice, can be associated with pain, swelling, and bleeding, while CO_2_ laser therapy showed fewer side effects. These results favor the use of CO_2_ laser therapy as an efficient and well-tolerated intervention. MAL-PDT and ALA-PDT with red light were associated with more frequent skin reactions, while other treatments such as diclofenac and IMB had either fewer or specific side effects. The qualitative analysis of the side effect makes a comparative analysis difficult. Thus, the choice of therapy depends on individual patient needs and preferences, as is the case for AK.

From a clinical perspective, purely ablative approaches such as the CO_2_ laser or ER:YAG laser offer the advantage of rapid lesion clearance and may be preferable in thicker or hyperkeratotic lesions where topical drugs may not penetrate sufficiently to achieve their full therapeutic effect. Nonetheless, procedural treatments such as laser devices or PDT require trained personnel and specialized equipment which may not be accessible in all healthcare settings. To this end, topical drug-mediated approaches such as imiquimod are easier to use and are usually reimbursed by healthcare providers.

Some limitations of this meta-analysis should be discussed and kept in mind for the interpretation of the results. First, substantial heterogeneity was present across studies, both methodologically and clinically. Study designs, treatment protocols, and quality varied, potentially affecting comparability. Second, outcome definitions for clearance and recurrence were not standardized and often relied on clinical rather than histological assessments. Specifically, the interpretation of PRR was limited by inconsistent follow-up durations and non-standardized outcome definitions. Most studies relied on clinical assessments without histological confirmation, which may bias recurrence estimates and hinder comparability between treatments. Most studies included here had follow-up periods of 12 to 18 months, with some extending up to 5 years. However, the exact timing of recurrences was rarely specified. Only a few studies reported recurrences at defined time points such as 6 or 12 months. Several studies lacked sufficient follow-up data, limiting their interpretability. Thus, longer and standardized follow-up is needed to better assess long-term treatment outcomes. Third, follow-up durations differed widely and were generally short, limiting insights into long-term effectiveness. Fourth, surgical treatments were underrepresented or lacked sufficient outcome data, precluding robust conclusions about their comparative value although surgical approaches are commonly performed for AC. Fifth, the use of GLMM in the single-arm meta-analyses, while methodologically sound and advantageous over conventional approaches [14], may overstate the precision of estimates in subgroups with few studies. Finally, evidence of publication bias were detected, which may have skewed results toward favorable outcomes. A comprehensive overview of methodological and analytical limitations is provided in Supplementary Table S5.

Although a quantitative synthesis was not possible, the findings on adverse events reveal some differences in the side effect profiles of ablative and conservative therapies. Ablative interventions, such as the Er:YAG laser, are highly effective but are frequently associated with immediate post-procedural side effects, including pain, swelling, and bleeding. MAL-PDT and ALA-PDT with red light may cause localized skin reactions (e.g., burning, erythema), impacting patient comfort. Other options, like diclofenac, are better tolerated but less effective regarding clearance, while IMB showed a high rate of side effects and is currently no longer available in Europe.

## 5. Conclusions

This study provides a thorough overview of reported efficacy outcomes for practice-relevant interventions for AC. The CO_2_ laser and imiquimod interventions showed the most favorable clearance and recurrence rates. Further high-quality RCTs are desirable for this common condition to improve the evidence base and support future guideline developments.

## Figures and Tables

**Table 1:** PICO scheme showing eligibility criteria and outcomes of interest. Abbreviations: RCT: randomized controlled trials, PDT: photodynamic therapy.

Population	Intervention	Comparison	Outcome	Trials
Patients with actinic cheilitis (clinical or histological confirmed) with all grades of dysplasia subgroups (grade 1,2,3)	Any relevant therapy including: - Surgical: vermilionectomy, cryotherapy, laser, electrodessication - Non-Surgical: 5-fluorouracil, imiquimod, diclofenac, conventional/daylight/patch PDT, chemical peeling, ingenol mebutate gel - combination of these therapies	placebo, vehicle-controlled, active control (in RCTs) no control in uncontrolled or retrospective studies/case series	At least one of the following efficacy outcomes: - Participant clearance rate (PCR) defined as the rate of participants who had all (100%) baseline lesions cleared - Participant recurrence rate (PRR) defined as the rate of participants that had relapsed lesions And/or safety outcomes: - Adverse events (with frequency)	- Randomized controlled trials - Controlled and uncontrolled clinical trials - Prospective observational studies - Retrospective studies (e.g., case series > 5 patients)

**Table 2:** Participant clearance rates (PCR) by treatment. Abbreviations: IMB: ingenol mebutate, MAL: methyl-aminolevulinate, ALA: 5-aminolevulinic acid, PDT: photodynamic therapy, and CI: confidence interval.

Treatment	PCR	95% CI	I^2^	Number of Studies
CO_2_ laser	0.97	[0.90; 0.99]	0%	5
Imiquimod	0.91	[0.81; 0.96]	0%	2
Er:YAG laser	0.86	[0.79; 0.92]	0%	2
MAL daylight PDT	0.83	[0.69; 0.91]	6%	4
ALA red light PDT	0.76	[0.63; 0.85]	0%	4
MAL red light PDT	0.7	[0.59; 0.80]	47%	8
Diclofenac	0.53	[0.41; 0.66]	2%	4
IMB	0.92	[0.06; 1.00]	0%	2

**Table 3:** Participant recurrence rates (PRR) by treatment. Abbreviations: MAL: methyl-aminolevulinate, ALA: 5-aminolevulinic acid, PDT: photodynamic therapy, and CI: confidence interval.

Treatment	PRR	95% CI	I^2^	Number of Studies
Imiquimod	0.0	[0.00; 0.60]	na	2
CO_2_ laser	0.02	[0.00; 0.12]	0%	4
MAL red light PDT	0.09	[0.04; 0.17]	18%	4
ALA red light PDT	0.1	[0.05; 0.20]	0%	3
Er:YAG laser	0.14	[0.08; 0.21]	0%	2
Diclofenac	0.0	[0.00; 0.18]	na	1
MAL daylight PDT	0.1	[0.01; 0.32]	na	1
5-FU 5%	0.17	[0.02; 0.48]	na	1

## Data Availability

All study-related data and statistical code are stored at the Uniklinikum Erlangen, Friedrich-Alexander-Universität Erlangen-Nürnberg (FAU), 91054 Erlangen, Germany, and are available upon reasonable request.

## Supplementary Materials

The following supporting information can be downloaded at: https://www.mdpi.com/article/10.3390/biomedicines13081896/s1, Figure S1: Preferred Reporting Items for Systematic Reviews and Meta-Analysis (PRISMA) checklist; Table S1: Search queries for the databases Medline, Embase (both via Ovid), and the Cochrane Library Central; Table S2: Baseline characteristics of all included studies [1,2,3,7,10,11,16,17,18,19,20,21,22,23,24,25,26,27,28,29,30,31,32,33,34,35,36,37,38,39,40,41,42,43,45] sorted by treatment group. Black stars indicate study qualities assessed using the NHLBI tool, while red stars signify studies evaluated with the RoB 2 tool. A **★** denotes a high risk of bias. Abbreviations: MAL: Methyl aminolevulinate, ALA: aminolevulinic acid, PDT: photodynamic therapy, LED: light emitting diode, ER:YAG: erbium-yttrium aluminum garnet, NA: not available, RCT: randomized controlled trial, 5-FU: 5-Fluorouracil, IMB: Imiquimod, AFL: ablative fractional laser; Table S3: Summary of Findings Table (GRADE) for the comparison of Er:YAG AFL MAL-PDT to MAL-PDT. Abbreviations: MAL: Methyl aminolevulinate, PDT: photodynamic therapy, ER:YAG: erbium-yttrium aluminum garnet, RCT: randomized controlled trial, AFL: ablative fractional laser; Table S4: Frequency of reported adverse events by intervention type, as documented in the included studies [1,2,3,7,16,17,18,19,21,22,24,25,26,27,28,32,34,36,37,38,40] (where available). Abbreviations: MAL: Methyl aminolevulinate, ALA: aminolevulinic acid, PDT: photodynamic therapy, ER:YAG: erbium-yttrium aluminum garnet, NA: not available, 5-FU: 5-Fluorouracil; Table S5: Detailed Study Limitations; Supplementary Material S1: Statistical Methods—GLMM Meta-analysis of Proportions [13,14].
