# Supplementary material for: Actinic Cheilitis: A Systematic Review and Meta-Analysis of Interventions, Treatment Outcomes, and Adverse Events
Source: Biomedicines. 2025 Aug 4;13(8):1896. doi: 10.3390/biomedicines13081896 (PMC12383482; doi:10.3390/biomedicines13081896)
Supplement: Supplementary file 1 [file biomedicines-13-01896-s001.zip › suppl_table_S1.pdf]

Supplementary Table S1: Search queries for the databases Medline, Embase (both via Ovid) and the Cochrane Library Central.

**Ovid MEDLINE(R) and Epub Ahead of Print, In-Process & Other Non-Indexed Citations, Daily and Versions(R) 1946 to August 27, 2024**<br>**N=320 (27.08.2024)**
1. Actinic keratosis of the lips.mp.<br>2. Angular cheilitis.mp.<br>3. Actinic prurigo.mp.<br>4. Cheilosis actinic.mp.<br>5. Lip keratosis.mp.<br>6. Cheilitis actinica acuta.mp.<br>7. Sailor* lip.mp.<br>8. Actinic ray.mp.<br>9. vermillionectomy.mp.<br>10. laser.mp. or exp Lasers/<br>11. photodynamic therapy.mp. or exp Photochemotherapy/<br>12. cryotherapy.mp. or exp Cryotherapy/<br>13. cryosurgery.mp. or exp Cryosurgery/<br>14. exp Electrosurgery/<br>15. imiquimod.mp. or exp Imiquimod/<br>16. diclofenac.mp. or exp Diclofenac/<br>17. 5-FU.mp. or exp Fluorouracil/<br>18. ingenol mebutate.mp.<br>19. lip shave.mp.<br>20. ALA.mp.<br>21. exp Aminolevulinic Acid/ or MAL.mp.<br>22. peeling.mp.<br>23. exp Keratolytic Agents/ or exp Trichloroacetic Acid/ or exp Chemexfoliation/ or chemical peeling.mp.<br>24. Jessner's solution.mp.<br>25. glycolic acid.mp.<br>26. solaraze.mp.<br>27. picato.mp.<br>28. aldara.mp.<br>29. zyclara.mp.<br>30. metvix.mp.<br>31. ameluz.mp.<br>32. BF-200 ALA.mp.<br>33. 9 or 10 or 11 or 12 or 13 or 14 or 15 or 16 or 17 or 18 or 19 or 20 or 21 or 22 or 23 or 24 or 25 or 26 or 27 or 28 or 29 or 30 or 31 or 32<br>34. Actinic cheilosis.mp.<br>35. exp Lip Neoplasms/ or actinic cheilitis.mp.<br>36. 1 or 2 or 3 or 4 or 5 or 6 or 7 or 8 or 34 or 35<br>37. 33 and 36
**Embase 1974 to 2020 December 11**<br>**N=1531 (14.12.2020)**
1. actinic cheilitis.mp.<br>2. lip neoplasm.mp. or exp lip tumor/<br>3. Actinic cheilosis.mp.<br>4. Actinic keratosis of the lips.mp.<br>5. Angular cheilitis.mp.

6. Actinic prurigo.mp. or exp actinic prurigo/
7. Cheilosis actinic.mp. or exp malignant transformation/
8. Lip keratosis.mp.
9. Sailor* lip.mp.
10. Actinic ray.mp.
11. 1 or 2 or 3 or 4 or 5 or 6 or 7 or 8 or 9 or 10
12. vermillionectomy.mp.
13. exp carbon dioxide laser/ or exp erbium YAG laser/ or laser.mp. or exp laser/
14. photodynamic therapy.mp. or exp photodynamic therapy/
15. cryotherapy.mp. or exp cryosurgery/ or exp cryotherapy/
16. Electrosurgery.mp. or exp electrosurgery/
17. exp imiquimod/ or imiquimod.mp.
18. exp diclofenac/ or diclofenac.mp.
19. 5-FU.mp. or exp fluorouracil/
20. ingenol mebutate.mp. or exp ingenol mebutate/
21. lip shave.mp.
22. ALA.mp.
23. Aminolevulinic Acid.mp. or exp aminolevulinic acid/
24. MAL.mp.
25. peeling.mp.
26. Trichloroacetic Acid.mp. or exp trichloroacetic acid/
27. chemical peeling.mp. or exp chemexfoliation/
28. Jessner's solution.mp.
29. glycolic acid.mp. or exp glycolic acid/
30. solaraze.mp.
31. picato.mp.
32. aldara.mp.
33. zyclara.mp.
34. metvix.mp. or exp aminolevulinic acid methyl ester/
35. ameluz.mp.
36. BF-200 ALA.mp.
37. 12 or 13 or 14 or 15 or 16 or 17 or 18 or 19 or 20 or 21 or 22 or 23 or 24 or 25 or 26 or 27 or 28 or 29 or 30 or 31 or 32 or 33 or 34 or 35 or 36
38. 11 and 37

Cochrane Library CENTRAL
N= 86 trials (27.08.2024)

#1 Actinic cheilitis
#2 Angular cheilitis
#3 Actinic ray
#4 Actinic prurigo
#5 Cheilosis actinic
#6 Lip keratosis
#7 Lip neoplasm
#8 Actinic cheilosis
#9 Actinic keratosis of the lips
#10 Sailor‘s lip
#11 precancerous lesion
#12 #1 or #2 or #3 or #4 or #5 or #6 or #7 or #8 or #9 or #10 or #11
#13 Vermilionectomy
#14 Photodynamic therapy
#15 Cryotherapy
#16 cryosurgery
#17 Electrodessication

#18	electrosurgery
#19	Imiquimod
#20	diclofenac
#21	chemical peeling
#22	ingenol mebutate
#23	laser
#24	Carbon dioxide laser
#25	YAG laser
#26	Fluorouracil
#27	Lip shave
#28	MAL
#29	ALA
#30	Methylaminolevulinic acid
#31	Aminolevulinic acid
#32	TCA
#33	Trichloracetic acid
#34	BF-200 ALA
#35	Ameluz
#36	Metvix
#37	Zyclara
#38	Aldara
#39	Picato
#40	Solaraze
#41	Glycolic acid
#42	Jessner‘s solution
#43	Keratolytic agents
#44	Chemexfoliation
#45	Aminolevulinic acid methyl ester
#46	#13 or #14 or #15 or #16 or #17 or #18 or #19 or #20 or #21 or #22 or #23 or #24 or #25 or #26 or #27 or #28 or #29 or #30 or #31 or #32 or #33 or #34 or #35 or #36 or #37 or #38 or #39 or #40 or #41 or #42 or #43 or #44 or #45
#47	#12 and #46
