# Supplementary material for: Actinic Cheilitis: A Systematic Review and Meta-Analysis of Interventions, Treatment Outcomes, and Adverse Events
Source: Biomedicines. 2025 Aug 4;13(8):1896. doi: 10.3390/biomedicines13081896 (PMC12383482; doi:10.3390/biomedicines13081896)
Supplement: Supplementary file 1 [file biomedicines-13-01896-s001.zip › suppl_table_S2.pdf]

Supplementary Table S2 Baseline characteristics of all included studies sorted by treatment group.

Study	Study design and quality	Biopsy taken pre and post treatment	Treatment procedure	N	Sex (female, male)	Mean age in years (range/ other information)	Continent
**5-FU 5%**							
E. Epstein, 1977 [1]	retrospective ★	pre: ✓ post: in 6 cases	three to four times daily	12	f: 25% m: 75%	56	Europe
**5% Imiquimod**							
T. Rosen, 2016 [2]	retrospective ★★	pre: ✓ post: ✓	once daily for a maximum of 8 weeks	50	f: 0% m: 100%	<50	North America
K. J. Smith, 2002 [3]	retrospective ★★	pre: ✓ post: x	three days per week for up to 6 weeks	15	f: 20% m: 80%	66.5 (41-83)	North America
**ALA-PDT**							
S. Radakovic, 2020 [4]	prospective ★★★	pre: in 8 cases post: in case of non-CR	light source: LED, 635 ± 9 nm light dose: 37 J/cm^2	21 (19) *	f: 61.9% m: 38.1%	68.5	Europe
S. Radakovic, 2017 [5]	retrospective ★★	pre: in 6 cases post: ✓	light source: LED, 630 ± 9 nm light dose: 37 J/cm^2	11	f: 81.8% m: 18.2%	70.1	Europe
E. Sotiriou, 2010 [6]	prospective ★★★	pre: ✓ post: ✓	20% 5-ALA cream light source: non-coherent light, 570-670 nm light dose: 40 J/cm^2 fluence rate: 80 mW/cm^2 2 sessions	40 (38) *2	f: 0% m: 100%	64.8 (median)	Europe
E. Sotiriou, 2008 [7]	retrospective ★★	pre: ✓ post: ✓	20% 5-ALA cream light source: non-coherent light, 570-670 nm light dose: 40 J/cm^2 fluence rate: 80 mW/cm^2	10	f: 0% m: 100%	65.4	Europe

			2 sessions				
**CO_2-Laser**							
S. Hohenleutner, 1999 [8]	prospective ★★	pre: ✓<br>post: NA	defocused CO_2 laser (10 W)<br>2-3 sessions	19	NA	NA	Europe
R. G. Dufresne, 1988 [9]	prospective ★	pre: in 8 cases<br>post: NA	Initial Laser Pass: defocused CO_2 laser (3-5 W, 2 mm spot)<br>Char Removal with hydrogen peroxide on a cotton-tip applicator<br>Targeted Pulsing: pulsed laser (0.05-0.1 s)<br>Use of a superpulsed CO_2 laser in some cases	13	f: 38.5%<br>m: 61.5%	67.0	North America
D. C. Whitaker,1987 [10]	prospective ★★	pre: ✓<br>post: ✓	Initial Laser Pass: defocused CO_2 laser (2 mm spot, 4-8 W) with an articulating arm at 133-256 W/cm² held 4-6 cm from the tissue<br>Char Removal: cleaning with a moist sponge<br>Targeted Retreatment: examine and retreat any remaining abnormal areas with the laser<br>Wound Sealing: second pass at a lower power (1-4 W) to seal the wound	16	f: 6.3%<br>m: 93.7%	66.0	North America
A. Neder, 1992 [11]	retrospective ★	pre: ✓<br>post: NA	Outline: continuous CO_2 laser (5 W)<br>Ablation: 8 W pulsed CO_2 laser in a crisscross pattern<br>Vaporization: 3 W continuous defocused beam	16	NA	NA	Asia
L. M. David, 1985 [12]	retrospective ★	pre: ✓<br>post: NA	Initial Laser Pass: continuous CO_2 laser (15 W, 3 mm spot)<br>Char Removal with hydrogen peroxide on a cotton-tip applicator<br>1-3 sessions	8	f: 25%<br>m: 75%	56.5	North America
**Daylight PDT**							
P. Martin-Carrasco, 2020 [13]	prospective ★	pre: NA<br>post: partly	MAL cream application without occlusion<br>Daylight exposure for 2h<br>Second session after two weeks	6	f: 16.7%<br>m: 83.3%	74.5	Europe
D. Andreadis, 2020 [14]	prospective ★★★	pre: ✓	MAL cream application (1 mm-thick layer, 160 mg/g)	22 (2	f: 15%<br>m: 85%	67.6 ± 9.4	Europe

		post: in case of non-CR	Daylight exposure for 2h<br>Second session after one week	0)<br>*3			
D. Fai, 2015 [15]	retrospective<br>★★	pre: in selected cases<br>post: NA	16% MAL cream application (0.5–1 mm layer, without occlusion)<br>Daylight exposure for 2h<br>second session after 1-2 weeks	10	f: 40%<br>m: 60%	74.7	Europe
A. Levi, 2019 [16]	retrospective<br>★★	pre: ✓<br>post: ✓	After Metvix® cream application (uncovered), exposure to sunlight for 2.5 h	11	f: 27.3%<br>m: 72.7%	59.2	Europe
**Diclofenac**							
M. Ulrich, 2011 [17]	prospective<br>★★	pre: ✓<br>post: NA	3% Diclofenac in 2.5% hyaluronic acid twice daily for 90 days	6	NA	NA	Europe
S. Lima Gda, 2010 [18]	prospective<br>★★	pre: NA<br>post: in case of non-CR	3% Diclofenac in 2.5% hyaluronic acid gel twice daily<br>for 30 to 180 days	27	f: 11.1%<br>m: 88.9%	58	South America
A. K. G. Gonzaga, 2018 [19]	prospective<br>★★★	pre: in 9 selected cases<br>post: NA	3% Diclofenac gel three times daily for 90 days	19	f: 21.1%<br>m: 78.9%	58.6	South America
C. Ulrich, 2007 [20]	retrospective<br>★★	pre: ✓<br>post: ✓	3% Diclofenac gel twice daily for 6 weeks	6	f: 50%<br>m: 50%	70.0	Europe
**Er:YAG Laser**							
A. Orenstein, 2007 [21]	retrospective<br>★★	pre: in 8 cases<br>post: NA	Laser Resurfacing: Er:YAG laser (3 mm spot, 1.2–1.7 J/pulse (16.97–24.05 J/cm²), 10 Hz)<br>Layer-by-layer application until capillary bleeding in the deep vermilion layer<br>Postoperative Care: lip dressing with 3% synthomycin antibiotic ointment and covering with ice-cold saline-soaked gauze	12	f: 41.7%<br>m: 58.3%	52.7	Asia
P. Armenores, 2010 [22]	retrospective<br>★★	pre: partly (91.9%)<br>post: NA	Laser Treatment: Er:YAG laser (4 mm spot), for one 100 µm ablative pass, followed by two passes combining 100 µm ablation and 100 µm coagulation<br>Postoperative Care: antibiotics, and saline washes every 2h, followed by application of mupirocin ointment or petroleum jelly	99	f: 31.3%<br>m: 86.7%	52.8	Australia
**IMB**							

R. C. Rossini, 2021 [23]	prospective ★★	pre: ✓ post: ✓	IMB gel 0.015% (Picato®; Leo Pharma) for self-application to the entire lower lip for three days	14	f: 35.7% m: 64.3%	69.4 ± 9.2	South America
A. Florez, 2017 [24]	retrospective ★	pre: in 3 cases post: NA	IMB 0.015% gel daily for 3 days	7	f: 42.9% m: 57.1%	72.9	Europe
**MAL-PDT**							
K. Dryk, 2014 [25]	prospective ★★	pre: ✓ post: ✓	Fractionated Illumination: 3 h after MAL cream application, irradiation of the area with red light at 20 J/cm², then application of a second red light dose of 80 J/cm² at 5 h	9	NA	NA	Europe
S. K. Kim, 2013 [26]	prospective ★★	pre: ✓ post: in case of CR	3 h after MAL cream application (with occlusive dressing), irradiation with red light (635 nm), with a dose of 37 J/cm²	10	NA	NA	Asia
J. A. Suarez-Perez, 2015 [27]	prospective ★★	pre: ✓ post: ✓	3 h after MAL cream application (160 mg/g, with occlusive dressing), irradiation with 20 J/cm² of red light (630 nm) Post-Treatment Care: Application of an occlusive dressing for 2 h, then delivery of a second light dose of 80 J/cm²	10	f: 20% m: 80%	66.1 ± 7.4	Europe
P. G. Calzavara-Pinton, 2013 [28]	retrospective ★★★	pre: ✓ post: NA	3-4 h after Metvix® cream application (1 mm thick layer, with occlusive dressing), irradiation with 37 J/cm² of red light (635 ± 18 nm) for one or more sessions	43	f: 37.2% m: 62.8%	71 ± 8	Europe
D. Fai, 2012 [29]	retrospective ★★	pre: in 6 cases post: NA	2-3 h after Metvix® cream application (160 mg/g, with an occlusive dressing), irradiation with red light source (Aktilite) at a dose of 37 J/cm²	29	f: 31.0% m: 60.0%	71.5	Europe
Y. N. Chaves, 2017 [30]	prospective ★★★	pre: ✓ post: ✓	3 h after Metvix® cream application (with plastic film and aluminum foil), irradiation of the lesion with red light (Aktilite) from 5-8 cm away, with a total dose of 37 J/cm² (irradiance of 71 mW/cm² for 8 minutes) Second session after two weeks	16	f: 37.5% m: 62.5%	64.5 (median)	South America
E. Sotiriou, 2011 [31]	prospective ★★	pre: ✓ post: ✓	3 h after Metvix® cream application (with an occlusive, light-impenetrable dressing), irradiation with red light (570–670 nm) at a light dose of 40 J/cm² and fluence rate of 80 mW/cm²	34 (30) *4	f: 2.9% m: 97.1%	65.26	Europe

			Second session after two weeks<br>Post-PDT Treatment: application of 5% imiquimod cream after two weeks over the entire lower lip once daily, 3 days per week for 4 weeks				
C. Berking, 2007 [32]	prospective ★★	pre: ✓<br>post: ✓	3 h after Metvix® cream application (with a self-adhesive polyethylene dressing and aluminum),<br>irradiation with red light (630nm, light dose: 37 J/cm², fluence rate: 68 mJ/cm²/s for 12 min, distance of 5–7 cm)<br>Second session after 1 week	15	f: 40%<br>m: 60%	68.0	Europe
**5-FU vs. chemical peel vs. lip shave vs. CO_2 laser**							
J. K. Robinson, 1989 [33]	RCT ★	pre: ✓<br>post: ✓	topical 5-FU 5%	10	f: 5%<br>m: 95%	48-70(range)	North America
		pre: ✓<br>post: ✓	chemical peel with trichloroacetic acid	10			
		pre: ✓<br>post: ✓	lip shave	10			
		pre: ✓<br>post: ✓	CO_2 laser	10			
**Er:YAG AFL PDT vs. MAL-PDT**							
S. H. Choi, 2015 [34]	RCT ★	pre: ✓<br>post: ✓	2940 nm Er:YAG laser with 300 µm ablation depth, level 1 coagulation, 22% density, and single pulse.<br>Thereafter: immediate 16% MAL cream application (1 mm layer with 5 mm margin).<br>Covering with occlusive dressing for 3 h.<br>Light source: LED lamp (632 nm)<br>light dose: 37 J/cm²	14 (13) *5	f: 35.7%<br>m: 64.3%	66.7 ± 8.7	Asia

		pre: ✓<br>post: ✓	Two sessions of conventional MAL-PDT one week apart	19 (17) *5	f: 42.1%<br>m: 57.9%	69.4 ± 6.9	
D.-Y. Ko, 2014 [35]	RCT ★	pre: ✓<br>post: ✓	Er:YAG laser with 550–600 μm laser ablation depth, level 1 coagulation, 22% treatment density, single pulse. Thereafter: MAL-PDT with LED (37 J/cm^2) 1 or 2 sessions	12	NA	NA	Asia
		pre: ✓<br>post: ✓	Conventional MAL-PDT: Application of MAL cream under occlusion for 3 h Irradiation with a red LED (37 J/cm^2) 1 or 2 sessions	37			
**Imiquimod vs. IMB vs. Diclofenac**							
H. Husein-ElAhmed, 2019 [36]	RCT ★	pre: NA<br>post: NA	Imiquimod 5% once daily for 3 days per week for 4 weeks	10	f: 53.3%<br>m: 46.7%	73.7	Europe
		pre: NA<br>post: NA	IMB 150 Ig/g gel (Picato®; Leo Pharma), daily for 3 days	10			
		pre: NA<br>post: NA	3% Diclofenac gel twice daily for 6 weeks	10			

Black stars indicate study qualities assessed using the NHLBI tool, while red stars signify studies evaluated with the RoB 2 tool. A ★ denotes a high risk of bias. N is the number of reported participants in each study. Abbreviations: MAL: Methyl aminolevulinate, ALA: aminolevulinic acid, PDT: photodynamic therapy, LED: light emitting diode, ER:YAG: erbium-yttrium aluminum garnet, NA: not available, RCT: randomized controlled trial, 5-FU: 5-Fluorouracil, IMB: Imiquimod, AFL: ablative fractional laser

* In this study, 21 patients were enrolled, but outcome data were available for only 19. Sex and age data refer to all 21 patients; the 19 with available outcome data were included in the meta-analysis.

*2 In this study, 40 patients were enrolled, but outcome data were available for only 38. Sex and age data refer to all 40 patients; the 38 with available outcome data were included in the meta-analysis.

*3 In this study, 22 patients were enrolled, but outcome data were available for only 20. Sex and age data refer to the 20 patients with available outcome data, who were also included in the meta-analysis.

*4 In this study, 34 patients were enrolled, but outcome data were available for only 30. Sex and age data refer to all 34 patients; the 30 with available outcome data were included in the meta-analysis.

*[5] In this study, 14 and 19 patients were enrolled in the two groups, but outcome data were available for only 13 and 17, respectively. Sex and age data refer to all enrolled patients (14 and 19); only the 13 and 17 patients with available outcome data were included in the meta-analysis.

1. Epstein, E., *Treatment of lip keratoses (actinic cheilitis) with topical fluorouracil.* Archives of Dermatology, 1977. **113**(7): p. 906-8.
2. Rosen, T., *Actinic cheilitis: Is Imiquimod 5% a viable treatment? A clinical study.* Australasian Journal of Dermatology, 2016. **57 (Supplement 2)**: p. 5.
3. Smith, K.J., et al., *Topical 5% imiquimod for the therapy of actinic cheilitis.* Journal of the American Academy of Dermatology, 2002. **47**(4): p. 497-501.
4. Radakovic, S., M. Dangl, and A. Tanew, *5-Aminolevulinic acid patch (Alacare) photodynamic therapy for actinic cheilitis: data from a prospective 12-month follow-up study on 21 patients.* Journal of the European Academy of Dermatology & Venereology, 2020. **34**(9): p. 2011-2015.
5. Radakovic, S. and A. Tanew, *5-aminolaevulinic acid patch-photodynamic therapy in the treatment of actinic cheilitis.* Photodermatology, Photoimmunology & Photomedicine, 2017. **33**(6): p. 306-310.
6. Sotiriou, E., et al., *Photodynamic therapy with 5-aminolevulinic acid in actinic cheilitis: an 18-month clinical and histological follow-up.* Journal of the European Academy of Dermatology & Venereology, 2010. **24**(8): p. 916-20.
7. Sotiriou, E., et al., *Actinic cheilitis treated with one cycle of 5-aminolaevulinic acid-based photodynamic therapy: report of 10 cases.* British Journal of Dermatology, 2008. **159**(1): p. 261-2.
8. Hohenleutner, S., M. Landthaler, and U. Hohenleutner, *[CO(2) laser vaporisation of actinic cheilitis].* Hautarzt, 1999. **50**(8): p. 562-5.
9. Dufresne, R.G., Jr., et al., *Carbon dioxide laser treatment of chronic actinic cheilitis.* Journal of the American Academy of Dermatology, 1988. **19**(5 Pt 1): p. 876-8.
10. Whitaker, D.C., *Microscopically proven cure of actinic cheilitis by CO2 laser.* Lasers in Surgery & Medicine, 1987. **7**(6): p. 520-3.
11. Neder, A., O. Nahlieli, and I. Kaplan, *CO 2 laser used in surgical treatment of actinic cheilitis.* Journal of Clinical Laser Medicine & Surgery, 1992. **10**(5): p. 373-5.
12. David, L.M., *Laser vermilion ablation for actinic cheilitis.* Journal of Dermatologic Surgery & Oncology, 1985. **11**(6): p. 605-8.
13. Martin-Carrasco, P., et al., *Actinic Cheilitis Treated With Daylight Photodynamic Therapy.* Actas Dermo-Sifiliograficas, 2020. **111**(10): p. 883-885.
14. Andreadis, D., et al., *Daylight photodynamic therapy for the management of actinic cheilitis.* Archives of Dermatological Research, 2020. **312**(10): p. 731-737.
15. Fai, D., et al., *Daylight photodynamic therapy with methyl-aminolevulinate for the treatment of actinic cheilitis.* Dermatologic Therapy, 2015. **28**(6): p. 355-68.
16. Levi, A., et al., *Daylight photodynamic therapy for the treatment of actinic cheilitis.* Photodermatology, Photoimmunology & Photomedicine, 2019. **35**(1): p. 11-16.
17. Ulrich, M., et al., *Non-invasive diagnosis and monitoring of actinic cheilitis with reflectance confocal microscopy.* Journal of the European Academy of Dermatology & Venereology, 2011. **25**(3): p. 276-84.
18. Lima Gda, S., et al., *Diclofenac in hyaluronic acid gel: an alternative treatment for actinic cheilitis.* Journal of Applied Oral Science, 2010. **18**(5): p. 533-7.
19. Gonzaga, A.K.G., et al., *Diclofenac sodium gel therapy as an alternative to actinic cheilitis.* Clinical Oral Investigations, 2018. **22**(3): p. 1319-1325.
20. Ulrich, C., et al., *Management of actinic cheilitis using diclofenac 3% gel: a report of six cases.* British Journal of Dermatology, 2007. **156 Suppl 3**: p. 43-6.
21. Orenstein, A., et al., *A new modality in the treatment of actinic cheilitis using the Er:YAG laser.* Journal of Cosmetic & Laser Therapy, 2007. **9**(1): p. 23-5.
22. Armenores, P., et al., *Treatment of actinic cheilitis with the Er:YAG laser.* Journal of the American Academy of Dermatology, 2010. **63**(4): p. 642-6.
23. Rossini, R.C., et al., *Ingenol mebutate treatment for actinic cheilitis: clinical, histopathological and p53 profile of 14 cases.* Journal of Dermatological Treatment, 2021. **32**(8): p. 1049-1052.
24. Florez, A., A. Batalla, and C. de la Torre, *Management of actinic cheilitis using ingenol mebutate gel: A report of seven cases.* Journal of Dermatological Treatment, 2017. **28**(2): p. 149-151.
25. Dryk, K., et al., *The efficacy of fractionated photodynamic therapy in actinic cheilitis of the lower lip.* Journal of the American Academy of Dermatology, 2014. **70**(5): p. AB157.
26. Kim, S., H. Song, and Y. Kim, *Treatment of actinic cheilitis with topical photodynamic therapy.* Journal of Investigative Dermatology, 2013. **133**: p. S179.
27. Suarez-Perez, J.A., et al., *Treatment of actinic cheilitis with methyl aminolevulinate photodynamic therapy and light fractionation: a prospective study of 10 patients.* European Journal of Dermatology, 2015. **25**(6): p. 623-4.
28. Calzavara-Pinton, P.G., M.T. Rossi, and R. Sala, *A retrospective analysis of real-life practice of off-label photodynamic therapy using methyl aminolevulinate (MAL-PDT) in 20 Italian dermatology departments. Part 2: oncologic and infectious indications.* Photochemical & Photobiological Sciences, 2013. **12**(1): p. 158-65.
29. Fai, D., et al., *Methyl-aminolevulinate photodynamic therapy for the treatment of actinic cheilitis: a retrospective evaluation of 29 patients.* Giornale Italiano di Dermatologia e Venereologia, 2012. **147**(1): p. 99-101.
30. Chaves, Y.N., et al., *Evaluation of the efficacy of photodynamic therapy for the treatment of actinic cheilitis.* Photodermatology, Photoimmunology & Photomedicine, 2017. **33**(1): p. 14-21.
31. Sotiriou, E., et al., *Sequential use of photodynamic therapy and imiquimod 5% cream for the treatment of actinic cheilitis: a 12-month follow-up study.* British Journal of Dermatology, 2011. **165**(4): p. 888-92.
32. Berking, C., et al., *The efficacy of photodynamic therapy in actinic cheilitis of the lower lip: a prospective study of 15 patients.* Dermatologic Surgery, 2007. **33**(7): p. 825-30.
33. Robinson, J.K., *Actinic cheilitis. A prospective study comparing four treatment methods.* Archives of Otolaryngology -- Head & Neck Surgery, 1989. **115**(7): p. 848-52.
34. Choi, S.H., K.H. Kim, and K.H. Song, *Efficacy of ablative fractional laser-assisted photodynamic therapy for the treatment of actinic cheilitis: 12-month follow-up results of a prospective, randomized, comparative trial.* British Journal of Dermatology, 2015. **173**(1): p. 184-91.
35. Ko, D.-Y., et al. *Ablative fractional laser-assisted methyl aminolaevulinate photodynamic therapy (MAL-PDT) vs. conventional MAL-PDT for the treatment of actinic cheilitis: a randomized, comparative study*. British journal of dermatology., 2014. **171**, 51-52 DOI: 10.1111/bjd.13282.
36. Husein-ElAhmed, H., F.M. Almazan-Fernandez, and S. Husein-ElAhmed, *Ingenol mebutate versus imiquimod versus diclofenac for actinic cheilitis: a 6-month follow-up clinical study.* Clinical & Experimental Dermatology, 2019. **44**(2): p. 231-234.
