# Supplementary material for: Actinic Cheilitis: A Systematic Review and Meta-Analysis of Interventions, Treatment Outcomes, and Adverse Events
Source: Biomedicines. 2025 Aug 4;13(8):1896. doi: 10.3390/biomedicines13081896 (PMC12383482; doi:10.3390/biomedicines13081896)
Supplement: Supplementary file 1 [file biomedicines-13-01896-s001.zip › suppl_table_S3.pdf]

Supplementary Table S3 Summary of Findings Table (GRADE) for the comparison of Er:YAG AFL MAL-PDT to MAL-PDT. Abbreviations: MAL: Methyl aminolevulinate, PDT: photodynamic therapy, ER:YAG: erbium-yttrium aluminum garnet, RCT: randomized controlled trial, AFL: ablative fractional laser

Er:YAG AFL MAL-PDT compared to MAL-PDT for cheilitis Actinica

Patient or population: cheilitis Actinica

Setting:

Intervention: Er:YAG AFL MAL-PDT

Comparison: MAL-PDT

Outcomes	Anticipated absolute effects* (95% CI)		Relative effect (95% CI)	№ of participants (studies)	Certainty of the evidence (GRADE)	Comments
	Risk with MAL-PDT	Risk with Er:YAG AFL MAL-PDT				
participant clearance rate (PCR) assessed with: % follow-up: mean 3 months	722 per 1.000	**928 per 1.000** (25 to 1.000)	**OR 4.97** (0.01 to 3197.20)	79 (2 RCTs)	⨁⨁⨁◯ Moderate[a]	
participant recurrence rate (PRR) assessed with: % follow-up: mean 12 months	278 per 1.000	**78 per 1.000** (23 to 240)	**OR 0.22** (0.06 to 0.82)	79 (2 RCTs)	⨁⨁⨁◯ Moderate[a]	

***The risk in the intervention group** (and its 95% confidence interval) is based on the assumed risk in the comparison group and the **relative effect** of the intervention (and its 95% CI).

CI: confidence interval; **OR:** odds ratio

GRADE Working Group grades of evidence
High certainty: we are very confident that the true effect lies close to that of the estimate of the effect.
Moderate certainty: we are moderately confident in the effect estimate: the true effect is likely to be close to the estimate of the effect, but there is a possibility that it is substantially different.
Low certainty: our confidence in the effect estimate is limited: the true effect may be substantially different from the estimate of the effect.
Very low certainty: we have very little confidence in the effect estimate: the true effect is likely to be substantially different from the estimate of effect.

Explanations

a. high risk of bias was assessed by the RoB 2 Tool
