# Supplementary material for: Actinic Cheilitis: A Systematic Review and Meta-Analysis of Interventions, Treatment Outcomes, and Adverse Events
Source: Biomedicines. 2025 Aug 4;13(8):1896. doi: 10.3390/biomedicines13081896 (PMC12383482; doi:10.3390/biomedicines13081896)
Supplement: Supplementary file 1 [file biomedicines-13-01896-s001.zip › suppl_table_S4.pdf]

Supplementary Table S4: Frequency of reported adverse events by intervention type, as documented in the included studies (where available). Abbreviations: MAL: Methyl aminolevulinate, ALA: aminolevulinic acid, PDT: photodynamic therapy, ER:YAG: erbium-yttrium aluminum garnet, NA: not available, 5-FU: 5-Fluorouracil.

**Intervention**	**Adverse events**	**Reported frequency**
5-FU 5%	Erosion	NA
	Pain	NA
5% Imiquimod	Erosion	NA
	Erythema	NA
	Induration	NA
	Ulceration	NA
ALA-PDT	Burning sensation	38/ 38 (100%) [1]
		10/10 (100%) [2]
	Blistering (single reported)	10/38 (26.3%) [1]
	Erythema (single reported)	26/38 (68.4%) [1]
		12/38 (31.6%) [1]
		10/10 (100%) [2]
	Herpes reactivation	5/11 (45.5%) [3]
	Haemorrhagic crusting	4/38 (10.5%) [1]
	Oedema (single reported)	24/38 (63.2%) [1]
	Oedema, blistering and erosions	17/21 (81%) [4]
	Pain	6/11 (54.5%) [3]

		38/38 (100%) [1]
	Severe reactions	4/21 (19%) [4]
	Swelling and blistering	2/10 (20%) [2]
CO_2-Laser	Focal linear scar	2/13 (15.4%) [5]
	Hypertrophic scar	2/19 (10.5%) [6]<br>1/13 (7.7%) [5]
	Pain	1/13 (7.7%) [5]
Daylight PDT	Erosions	2/20 (10%) [7]
	Erythema and/or Edema	6/10 (60%) [8]
	Erythema, oedema and mild scaling	16/20 (80%) [7]
	Pain	3/6 (50%) [9]<br>5/10 (50%) [8]
Diclofenac in hyaluronic acid gel	Allergic reaction -> edema and intense	2/26 (7.7%) [10]
	Erythema -> burning sensation	NA
Diclofenac sodium gel	Erythema, burning sensation, itching	5/19 (26.3%) [11]
Er:YAG Laser	Bleeding	5/12 (41.7%) [12]<br>52/77 (67.5%) [13]

	Burning	14/77 (18.2%) [13]
	Cracking	14/77 (18.2%) [13]
	Dryness	10/77 (18.2%) [13]
	Dysesthesias	8/77 (10.4%) [13]
	Edema	7/12 (58.3%) [12]
	Erythema	37/77 (48.1%) [13]
	Exudation	42/77 (54,8%) [13]
	Infection	1/12 (8.3%) [12]
	Pain	4/12 (33%) [12]<br>58/77 (75.3%) [13]
	Parasthesia	4/12 (33%) [12]
	Pruritus	4/77 (5.2%) [13]
	Pyogenic granuloma	1/12 (8.3%) [12]
	Swelling	43/77 (55.8%) [13]
Ingenol mebutate	Erosion/ulceration	10/14 (71%) [14]
	Erythema	14/14 (100%) [14]

	Leukoplakia<br><br>Pain/burn sensation<br><br>Scaling	14/14 (100%) [14]<br><br>3/14 (21%) [14]<br><br>6/14 (43%) [14]
MAL-PDT	Blisters<br><br>Blistering crusting<br><br>Burning sensation<br><br><br>Crusting<br><br>Edema<br><br><br><br><br><br><br>Erosions／superficial ulcerations<br><br>Erythema	3/10 (30%) [15]<br><br>6/30 (20%) [16]<br><br>30/30 (100%) [16]<br>12/30 (40%) [16]<br><br>25/30 (83%) [16]<br><br>8/10 (80%) [15]<br>19/29 (65.5%) [17]<br>24/30 (80%) [16]<br>21/30 (70%) [16]<br>5/11 (45.5%) [18]<br>4/15 (26.7%) [19]<br><br>14/30 (47%) [16]<br><br>10/10 (100%) [15]<br>24/29 (82.8%) [17]<br>30/30 (100%) [16]<br>30/30 (100%) [16]<br>11/11 (100%) [18]<br>5/15 (33%) [19]

	Erosions or ulcerative lesions	16/29 (55.2%) [17]<br>26/29 (89.7%) [17]
	Itching	14/30 (47%) [16]
	Hemorrhagic crusts	9/10 (90%) [15]
	Local inflammatory reaction	18/43 (41.9%) [12]
	Pain	5/43 (11.6%) [20]<br>30/30 (100%) [16]<br>4/11 (36.4%) [18]
	Recurrence of herpes labialis	1/23 (4.3%) [21]
	Scaling	25/30 (83%) [16]
	Superficial desquamation and hemorrhagic crusting	15/15 (100%) [19]

1. Sotiriou, E., et al., *Photodynamic therapy with 5-aminolevulinic acid in actinic cheilitis: an 18-month clinical and histological follow-up.* Journal of the European Academy of Dermatology & Venereology, 2010. **24**(8): p. 916-20.
2. Sotiriou, E., et al., *Actinic cheilitis treated with one cycle of 5-aminolaevulinic acid-based photodynamic therapy: report of 10 cases.* British Journal of Dermatology, 2008. **159**(1): p. 261-2.
3. Radakovic, S. and A. Tanew, *5-aminolaevulinic acid patch-photodynamic therapy in the treatment of actinic cheilitis.* Photodermatology, Photoimmunology & Photomedicine, 2017. **33**(6): p. 306-310.

4. Radakovic, S., M. Dangl, and A. Tanew, *5-Aminolevulinic acid patch (Alacare) photodynamic therapy for actinic cheilitis: data from a prospective 12-month follow-up study on 21 patients.* Journal of the European Academy of Dermatology & Venereology, 2020. **34**(9): p. 2011-2015.
5. Neder, A., O. Nahlieli, and I. Kaplan, *CO 2 laser used in surgical treatment of actinic cheilitis.* Journal of Clinical Laser Medicine & Surgery, 1992. **10**(5): p. 373-5.
6. Hohenleutner, S., M. Landthaler, and U. Hohenleutner, *[CO(2) laser vaporisation of actinic cheilitis].* Hautarzt, 1999. **50**(8): p. 562-5.
7. Andreadis, D., et al., *Daylight photodynamic therapy for the management of actinic cheilitis.* Archives of Dermatological Research, 2020. **312**(10): p. 731-737.
8. Fai, D., et al., *Daylight photodynamic therapy with methyl-aminolevulinate for the treatment of actinic cheilitis.* Dermatologic Therapy, 2015. **28**(6): p. 355-68.
9. Martin-Carrasco, P., et al., *Actinic Cheilitis Treated With Daylight Photodynamic Therapy.* Actas Dermo-Sifiliograficas, 2020. **111**(10): p. 883-885.
10. Lima Gda, S., et al., *Diclofenac in hyaluronic acid gel: an alternative treatment for actinic cheilitis.* Journal of Applied Oral Science, 2010. **18**(5): p. 533-7.
11. Gonzaga, A.K.G., et al., *Diclofenac sodium gel therapy as an alternative to actinic cheilitis.* Clinical Oral Investigations, 2018. **22**(3): p. 1319-1325.
12. Orenstein, A., et al., *A new modality in the treatment of actinic cheilitis using the Er:YAG laser.* Journal of Cosmetic & Laser Therapy, 2007. **9**(1): p. 23-5.
13. Armenores, P., et al., *Treatment of actinic cheilitis with the Er:YAG laser.* Journal of the American Academy of Dermatology, 2010. **63**(4): p. 642-6.
14. Rossini, R.C., et al., *Ingenol mebutate treatment for actinic cheilitis: clinical, histopathological and p53 profile of 14 cases.* Journal of Dermatological Treatment, 2020: p. 1-4.
15. Suarez-Perez, J.A., et al., *Treatment of actinic cheilitis with methyl aminolevulinate photodynamic therapy and light fractionation: a prospective study of 10 patients.* European Journal of Dermatology, 2015. **25**(6): p. 623-4.
16. Sotiriou, E., et al., *Sequential use of photodynamic therapy and imiquimod 5% cream for the treatment of actinic cheilitis: a 12-month follow-up study.* British Journal of Dermatology, 2011. **165**(4): p. 888-92.
17. Fai, D., et al., *Methyl-aminolevulinate photodynamic therapy for the treatment of actinic cheilitis: a retrospective evaluation of 29 patients.* Giornale Italiano di Dermatologia e Venereologia, 2012. **147**(1): p. 99-101.
18. Levi, A., et al., *Daylight photodynamic therapy for the treatment of actinic cheilitis.* Photodermatology, Photoimmunology & Photomedicine, 2019. **35**(1): p. 11-16.

19. Berking, C., et al., *The efficacy of photodynamic therapy in actinic cheilitis of the lower lip: a prospective study of 15 patients.* Dermatologic Surgery, 2007. **33**(7): p. 825-30.
20. Calzavara-Pinton, P.G., et al., *A retrospective analysis of real-life practice of off-label photodynamic therapy using methyl aminolevulinate (MAL-PDT) in 20 Italian dermatology departments. Part 2: oncologic and infectious indications.* Photochemical & Photobiological Sciences, 2013. **12**(1): p. 158-65.
21. Chaves, Y.N., et al., *Evaluation of the efficacy of photodynamic therapy for the treatment of actinic cheilitis.* Photodermatology, Photoimmunology & Photomedicine, 2017. **33**(1): p. 14-21.
