# Supplementary material for: Actinic Cheilitis: A Systematic Review and Meta-Analysis of Interventions, Treatment Outcomes, and Adverse Events
Source: Biomedicines. 2025 Aug 4;13(8):1896. doi: 10.3390/biomedicines13081896 (PMC12383482; doi:10.3390/biomedicines13081896)
Supplement: Supplementary file 1 [file biomedicines-13-01896-s001.zip › suppl_Table_S5.pdf]

Supplementary Table S5: Detailed Study Limitations

No.	Limitation	Description
1	**Small-study and publication bias**	Funnel plot asymmetry indicated potential small-study effects; results may be skewed toward favorable outcomes due to underreporting of negative findings.
2	**Methodological heterogeneity**	Studies varied in design (prospective vs. retrospective) and quality, limiting comparability.
3	**Clinical heterogeneity**	Treatment protocols differed within intervention groups, complicating pooled analyses.
4	**Precision inflation in GLMMs**	While GLMMs improve over traditional methods, they may inflate precision in subgroups with few studies.
5	**Inconsistent outcome definitions**	Clearance and recurrence were often defined clinically rather than histologically, limiting standardization.
6	**Short and variable follow-up**	Follow-up durations varied and were often too short to assess long-term efficacy or recurrence.
7	**Underrepresentation of surgical treatments**	Surgical modalities lacked sufficient data for inclusion, limiting conclusions about their effectiveness.
8	**Limited interpretability of heterogeneity metrics in small groups**	In treatment groups with few studies (e.g., n=2), I^2 statistics may misleadingly suggest low heterogeneity. In such cases, τ^2 offers a more accurate estimate of between-study variance.
