# Supplementary material for: Actinic Cheilitis: A Systematic Review and Meta-Analysis of Interventions, Treatment Outcomes, and Adverse Events
Source: Biomedicines. 2025 Aug 4;13(8):1896. doi: 10.3390/biomedicines13081896 (PMC12383482; doi:10.3390/biomedicines13081896)
Supplement: Supplementary file 1 [file biomedicines-13-01896-s001.zip › Supplementary Material S1.pdf]

Supplementary Material S1: Statistical Methods – GLMM Meta-analysis of Proportions

For the random-effects meta-analysis of proportions conducted in this study, we opted for a one-step approach using the GLMM rather than the conventional two-step methods (e.g., log, logit, arcsine, or Freeman–Tukey double-arcsine transformations and their back-transformations). The GLMM approach fully incorporates within-study uncertainties, which is particularly important when dealing with small sample sizes or extreme proportions, as observed in our study. Additionally, this method does not rely on a normality assumption, which may be inappropriate in this context, nor does it require corrections for zero counts[1].

In GLMM, the standard normal likelihood used in within-study modeling is replaced by a binomial likelihood:

$$L_i(\theta_i) = \frac{\exp(\theta_i)^{Y_i}}{(1+\exp(\theta_i))^{n_i}} \text{ ,}$$

where Y_i represents the observed event count, n_i is the sample size, and θ_i denotes the latent true proportion of study i [2]. After directly modelling event counts with binomial likelihoods, a specific link function is used to transform latent true proportions to a linear scale. Here we employed the logit transformation $g(\widehat{\theta_i}) = \log\left[\frac{\widehat{\theta_i}}{1-\widehat{\theta_i}}\right]$ where $\widehat{\theta_i}$ is the estimated θ_i. The logit transformation is the default link function in GLMM and is particularly suited for the data structure in our study. The binomial distribution with homogeneity assumption was used to calculate the pooled proportion for PRR when all arms showed zero recurrences because it was not possible to calculate a random effects model within GLMM.

1. Lin, L. and H. Chu, *Meta-analysis of Proportions Using Generalized Linear Mixed Models.* Epidemiology, 2020. **31**(5): p. 713-717.
2. Stijnen, T., T.H. Hamza, and P. Özdemir, *Random effects meta-analysis of event outcome in the framework of the generalized linear mixed model with applications in sparse data.* Stat Med, 2010. **29**(29): p. 3046-3067.
